# Adapting telehealth to address health equity: Perspectives of primary care providers across the United States

**DOI:** 10.1177/1357633X241238780

**Published:** 2024-03-22

**Authors:** Rachel Azar, Rachel Chan, Miriam Sarkisian, Robert D Burns, James P Marcin, Christine Gotthardt, Keshia R De Guzman, Jennifer L Rosenthal, Sarah C Haynes

**Affiliations:** 1 8789 UC Davis School of Medicine, Sacramento, CA, USA; 2 Department of Pediatrics, 8789 UC Davis Health, Sacramento, CA, USA; 3 Center for Health and Technology, 8789 UC Davis Health, Sacramento, CA, USA; 4 104824 School of Pharmacy, 1974 The University of Queensland, Brisbane, Australia; 5 430948 Centre for Online Health, 1974 The University of Queensland, Brisbane, Australia

**Keywords:** Telehealth, equity, vulnerable populations, primary care, pandemic‌

## Abstract

**Background:**

Telehealth has the potential to increase access to care for medically underserved patients. This qualitative study aimed to identify telecare practices used during the COVID-19 pandemic to meet the needs of patients experiencing homelessness, patients with disabilities, and patients with language preference other than English (LOE).

**Methods:**

We conducted a secondary qualitative data analysis of 47 clinician interviews at Federally Qualified Health Centers (FQHCs) around the country. Using thematic analysis, transcripts were coded by line-by-line by five qualitative researchers. A multidisciplinary team of telehealth experts, researchers and primary care clinicians reviewed memos and excerpts to generate major themes.

**Results:**

We identified six main areas demonstrating how community providers developed strategies or practices to improve access to care for vulnerable patients: reaching patients experiencing homelessness, serving deaf and hard of hearing patients, improving access for patients with disabilities, serving patients with LOE, improving access for mental and behavioral health services, and educating patients about telehealth. During the pandemic, FQHCs developed innovative solutions to provide access to care for the unhoused, including using telehealth in shelters, vans, and distributing devices like mobile phones and tablets. Telehealth reduced transportation burdens for patients with disabilities and reduced no-show rates for mental health services by adapting group therapy via telehealth features (like break-out rooms) and increasing provider capacity.

**Conclusion:**

Our study identified strategies adopted by FQHCs to serve underserved populations during the COVID-19 pandemic. Our findings highlight the need for enduring strategies to improve health equity through telehealth..

## Introduction

Telehealth has the potential to increase access to care by addressing factors such as lack of transportation, physical mobility challenges, and limited availability of healthcare.^1–4^ However, it is recognized that unless there is a specific focus on equity, interventions are likely to be implemented in a way that benefits those who already have relative advantages.^5^ Thus, to avoid widening disparities, it is of critical importance that implementation of telehealth includes attention to expanding access for the most vulnerable patients.^6,7^ The rapid rollout of telehealth during the COVID-19 pandemic demonstrated the importance of focusing on equity; patients with higher health literacy and digital literacy, higher income, flexible work arrangements, from urban areas, and with ability to speak English were more likely to successfully access telehealth services.^8–13^ Identifying evidence-based practices and understanding how these practices can be implemented into clinical practice and scaled are the next steps towards addressing meeting this challenge.

Federally Qualified Health Centers (FQHCs) are outpatient healthcare organizations that serve the uninsured, underinsured, and some of the most vulnerable and medically underserved patients in the United States. FQHCs across the country adopted telehealth during the COVID-19 pandemic to help meet patient needs as regulatory barriers were lifted. The knowledge generated by FQHCs around telehealth implementation during this period could be valuable for improving outreach and access for low-income patients, especially the most vulnerable patient populations, including people who are unhoused, people with disabilities, and people with a language preference other than English (LOE). To capture this knowledge, we conducted a secondary qualitative data analysis of in-depth interviews with primary care providers at FQHCs across the United States. The objectives of this study were to (a) identify the telehealth practices adopted by FQHCs to reach vulnerable populations, including patients who are unhoused and those with disabilities; (b) explore the practices utilized in delivering telehealth services to patients with LOE; and (c) identify potential barriers and facilitators of health equity within telehealth provision.

## Methods

### Participants and setting

The California Telehealth Resource Center (CTRC) is one of 12 federally-funded regional centers in the United States and U.S. Territories that serve as resources for telehealth education, expertise, and implementation guidance for healthcare providers, health systems, clinics, and government agencies.^14^ In partnership with eight other regional telehealth resource centers, the CTRC conducted in-depth interviews with providers, administrators, and IT staff at FQHCs around the country with the goal of informing future programming and resource development. In these interviews, respondents described how their center had adopted telehealth, including practices that had worked well, barriers faced and how they were addressed, and main challenges moving forward. Interviews were completed by CTRC staff and lasted approximately 60 minutes. Because we were specifically interested in how telehealth was used in direct patient care, we only included interviews with healthcare providers in this secondary analysis. These data consisted of 47 interviews completed with providers at 47 different FQHCs served by one of eight telehealth resource centers in 29 states. Participants included 24 physicians, 15 advanced practitioners (nurse practitioners and physician assistants), and 8 behavioral health providers (psychologists or therapists).

### Data analysis

Transcripts were analyzed using thematic analysis. We used Dedoose qualitative software^15^ to conduct our analysis.. We used a combination inductive/deductive approach to allow for identification of new important themes while also answering our specific questions related to how clinics addressed providing telehealth care to vulnerable patient populations and those with LOE. Five coders trained in qualitative research methods met in person to code the first two transcripts together and generate initial inductive codes and determine additional a priori deductive codes based on specific research questions related to vulnerable groups (e.g., caring for patients experiencing homelessness). Following this meeting, all five coders used Dedoose to independently code, with at least two coders coding each transcript to enhance reliability, reduce individual bias, and clarify coding decisions. Researchers followed Glaser's constant comparative method, in which each new line is compared to previous codes.^16^ Each coder also completed memos after coding each transcript. After all documents were coded, excerpts were extracted by code into a word document, where themes were grouped together, organized, and reviewed by the larger research team alongside each coder's memos.

### Ethical considerations

This study was a secondary data analysis of deidentified transcripts, it was classed and approved as “not human subjects research” by the UC Davis IRB.

## Results

We identified six main themes related to ways in which community providers developed strategies or practices to improve access to care for some of the most vulnerable patients. These themes include: (a) adopting telehealth strategies to meet the needs of unhoused patients, (b) reducing transportation burden on patients with disabilities and mobility issues and their families, (c) adopting strategies to serve deaf and hard of hearing people through telehealth, (d) providing telehealth for patients with LOE, (e) using telehealth to improve access to mental/behavioral health services, and (f) educating patients on telehealth to promote equity.

### Adopting telehealth strategies to meet the needs of unhoused patients

Prompted by the pandemic and a lifting of regulatory restrictions, FQHCs developed new strategies for ensuring access to care for unhoused patients. Several participants described setting up telehealth capacity in shelters or other community-based organizations that serve unhoused people. Using a designated telehealth room or computer in these spaces, patients could receive care from their primary care physicians and see specialists when needed. Another strategy adopted by community health centers was to set up telehealth capabilities in vans that were engaged in other outreach activities, such as HIV and hepatitis C screening and syringe exchange programs. This allowed providers to identify patients through outreach and connect them to a primary care or behavioral health provider, depending on their needs. Finally, numerous participants mentioned that their clinic had mitigated potential access issues by providing smartphones and tablets to unhoused patients who did not have one of these devices using channels such as shelters and outreach efforts. Participants also noted challenges with providing telehealth access to unhoused patients, citing issues with reliable cell phone service and internet. Many stated that unhoused patients often relied on telephone for completing visits.

### Reducing transportation burden on patients with disabilities and mobility issues and their families

Many participants highlighted the benefit of telehealth for patients who have disabilities that make transportation to a clinic visit difficult. One participant described,

“In-person visits are incredibly burdensome for people who are essentially homebound and seem unnecessary and almost mean to drag somebody who say is wheelchair bound or non-verbal in for an appointment when it's really you just need to talk to a caregiver.”

Others echoed this sentiment, expressing that while transportation is difficult for many low-income patients seen in these clinics, it is even more challenging for patients with disabilities. Telehealth services that allow the patient to be seen in their home in the company of a caregiver can improve quality of life by reducing the intense burden of travel. There may also be a benefit to seeing these patients in their home environment. Another provider stated,

“There was an older lady who…unfortunately had a stroke. And so that made her mobility a little bit more difficult and so I’ve done multiple video visits with her since. And I think it makes it easier for her son because he doesn’t have to fight with her to get her to the office. I can see her where she lives. And I think I had a better idea of exactly what she needed, better than I would have gotten in an in-person visit.”

### Strategies to serve deaf and hard of hearing patients through telehealth

Some FQHC providers were able to adapt to meet the needs of deaf and hard of hearing patients through the use of American Sign Language (ASL) over video, sending hearing amplifiers to patients that required them, and recommending specific headphones for video visits that have higher volume limits. However, meeting the needs of these patients was often challenging. Poor video quality was an especially important concern for these patients because unsuccessful video visits were not easily converted to audio. One participant expressed,

“I always try to use the video for all my deaf patients, but the quality of the video limits that sometimes. I often start on video and we have to hang up and use a relay service because the sign language, if its ghosting or choppy, we can’t understand each other.”

Finding ASL interpreters over telehealth was cited as a challenge by numerous providers.

### Providing telehealth for patients with a language preference other than English

There was large variability in providers’ perception of their ability to access interpreter services effectively and efficiently over telehealth. Several participants expressed concern about the ability of the clinic platform to appropriately handle requests for interpreters. Difficulty conducting a visit over video may have led to a higher number of patients with LOE being asked to come in to the clinic rather than use the telehealth option. One provider stated,

“If there weren’t family members there to help them, then it would be very difficult. So in those situations, we would bring patients into the clinic.”

However, many other providers expressed that they were able to meet the needs of patients with LOE using available interpreter services. Some clinics had developed workflows to seamlessly integrate interpreter services; others were still struggling to find solutions to this problem.

### Using telehealth to improve access to mental/behavioral health services

Mental and behavioral health providers perceived telehealth services as a key strategy for improving access for patients needing these often-scarce services. Numerous providers mentioned that telehealth had reduced no-show rates for behavioral health and had increased patient adherence to recommended behavioral health visits. One reason given for this is the potential for telehealth to mitigate anxieties that can surround in-person visits. One participant stated,

“The ability to be seen and heard without having to go into the world and face some of their anxiety or be able to talk to them while they are in their environment and not have to impose our environment on them. So we’re looking to really keep up with the telehealth.”

Another similarly mentioned,

“I think it's worked really well for our behavioral health, being able to talk to folks online. I think in such a small community, there is stigma when you walk into that side of the building and people see you.”

Several providers also mentioned that telehealth visits allowed them to see more patients than they otherwise might have been able to in person—at least in part due to lower no-show rates and increased availability of providers due to flexible and remote work arrangements—thus increasing access to necessary care.

### Improving visit quality by educating patients in telehealth processes

FQHC providers developed ways to educate patients in telehealth procedures to improve visit quality and promote equity by reaching patients that may have a harder time accessing telehealth services. These practices included providing support for setting up video applications needed for telehealth, walking patients through processes such as logging into their patient portal, testing their audio and video function, and practicing proper telehealth etiquette. One provider noted,

“We’ve done a lot of support for patient phone calls, talking them through, setting the app up to do telehealth visits from home…and just reminders, calls about how to log in before the appointment to make sure that they are logging in fine and not having connectivity issues.”

Several mentioned that having support resources designated for telehealth visits can improve the quality of visits by helping patients overcome technological barriers, particularly for those who have not previously used telehealth. As one participant stated,

“The way that we solve the problem is we have dedicated resources in the form of care coordinators that are super skilled at getting people comfortable laughing their way through the fumbles and the bumbles of getting it to happen, getting them set up if necessary, teeing them up.”

## Discussion

Our analysis highlights the telehealth strategies employed by FQHCs to meet the needs of some of the most vulnerable patients during the COVID-19 pandemic. Given that these community health centers are often under-resourced, it is unsurprising that they identified creative solutions to provide care to their most vulnerable patients, including the unhoused, patients requiring interpreter services, deaf or hard of hearing patients, patients with disabilities, and those utilizing mental health services. Identifying these strategies is important for understanding how telehealth could be implemented in a way that promotes health equity.

Our findings are especially important given that recent studies have highlighted disparities in how telehealth is accessed and used. Previous studies have found that telehealth users tend to have commercial insurance, speak English proficiently, and come from higher-resourced communities.^8,11,12,17–21^ Although most unhoused people own mobile phones,^22^ there remain significant challenges in their ability to access accessing telehealth care, including digital literacy, battery life, breakages, theft, and mistrust of the medical system.^14–16,21^ These patients thus more likely to miss visits, which may lead to less preventative care, higher utilization of the emergency department, and worse health outcomes.^17^ Several of the strategies identified by FQHC providers in our study have been utilized in the past, including mobile outreach vans, outreach at shelters, and partnerships with shelters and community organizations.^18–21^ Continued exploration into how these strategies could be adapted to improve access to primary care in this population is warranted.

Previous research has demonstrated lower access to telehealth services by patients with LOE.^7,12,19,23,24^ Numerous studies site similar barriers to the those identified by clinicians in our study, including technology-related barriers for LOE families, challenges integrating interpreter services into telehealth workflows,^25^ lack of knowledge around availability of interpreter services, and provider and patient discomfort with interpretation services over telehealth.^4,13,^^26^ Use of patient portals has been found to be lower among patients with LOE; therefore, telehealth services that rely on patient portals are likely less likely to have high adoption among this population.^26–29^ Deaf and hard of hearing patients have also been shown to have lower telehealth utilization, with many of the same reasons cited as challenges.^30,31^ Telehealth implementation frequently necessitates significant alterations in practices workflow yet the intricacies and depth of this transformation are commonly underestimated.^32^ Our study indicates that a multipronged approach that addresses digital literacy, health literacy, structural factors, and social factors may be helpful for addressing these barriers. Finally, telehealth may have the ability to improve access to care for persons with disabilities by mitigating challenges related to transportation and caregiving.^33,34^ More research assessing the effectiveness and implementation of specific strategies is needed to ensure optimal virtual care for these patients and their families.

For each population discussed in our findings, knowledge of telehealth and available services is essential for ensuring access to care. Telehealth education, outreach, and support by clinic staff could help to improve reach of telehealth services and related interventions. Previous research has found that strategies such as pre-visit telephone calls to discuss basics of telehealth appointments, recommendations for setting up the visit environment, education about technical requirements, and practice visits, can result in more successful telehealth visits.^35,36^ Clinics should ensure that all patients have access to these valuable educational resources. Equally significant is the extension of telecare education to healthcare providers. In a comprehensive study investigating the response of primary care in Australia to the COVID-19 pandemic using digital health strategies by reviewing 29 relevant papers, it was found that although the responses heavily relied on telehealth, mobile applications, and national hotlines to facilitate telecare services, a primary barrier was workforce training.^37^ This emphasizes the need for innovative virtual care strategies aimed at enhancing access, integration, safety, and quality within primary care settings. Ensuring the sustainability of telehealth beyond COVID-19 requires innovative approaches, such as adopting a “telehealth champion” model (people who advocate for telehealth and encourage others to try it), facilitating coordination, and fostering a strong willingness to practice telehealth.^38^

The most significant strength of our study was the inclusion of data from 47 clinics serving large underserved populations throughout different regions of the United States, promoting generalizability of our findings to other low-income and vulnerable communities. National-level research in this area is scarce and we benefited from having existing qualitative data from a previous improvement initiative. However, our study also has several limitations. Because this was a secondary analysis, we were not able to prompt respondents specifically on strategies for meeting the needs of vulnerable patient populations. This limits the available information in our interviews to strategies that were mentioned during general discussion about providing care to patients over telehealth. Second, we were unable to associate responses with provider or clinic characteristics, which could have given us more insight into how these characteristics may have facilitated rollout of specific strategies during the pandemic. We also did not have data on which clinics provided telehealth-only versus in-person services during the beginning of the pandemic, which likely affected patients’ use of telehealth. Finally, the strategies described by participants in this study were designed for a pandemic environment, during which resource availability, provider attitudes, and patient priorities likely differed from today. These strategies thus must be adapted to the current context of a post-pandemic world. Despite these limitations, our findings provide insight into ways that telehealth can be adapted to meet the needs of all patients and improve health equity.

### Conclusion

Our study highlights the importance of developing interventions and workflows that will ensure access to telehealth services for all patients, including the most vulnerable patients. Primary care providers have a wealth of knowledge that can serve as the building blocks for effective strategies; this knowledge should continue to be harnessed to ensure that strategies are fit to context. Combining telehealth with outreach strategies (e.g., such as screening and preventative services, syringe exchange programs, services for unhoused persons, services for immigrant and refugee populations) may be an effective way to improve access to care to these vulnerable patient populations. Exploration of implementation and dissemination strategies is needed to ensure that telehealth services reach the patients who have with the highest need for them.
